# Phosphate Solubilization and Plant Growth Promotion by *Pantoea brenneri* Soil Isolates

**DOI:** 10.3390/microorganisms11051136

**Published:** 2023-04-27

**Authors:** Aliya Suleimanova, Daria Bulmakova, Lidiya Sokolnikova, Evgenia Egorova, Daria Itkina, Olga Kuzminova, Albina Gizatullina, Margarita Sharipova

**Affiliations:** 1 Institute of Fundamental Medicine, Kazan Federal University, Kremlevskaya St. 18, 420008 Kazan, Russia; 2 Federal Research Center «Kazan Scientific Center of Russian Academy of Sciences», Lobachevskogo St. 2/31, 420111 Kazan, Russia

**Keywords:** phosphate solubilizing bacteria, *P. brenneri*, organic acids, PGPR, phosphorus

## Abstract

Phosphate solubilizing microorganisms (PSMs) in soil have been shown to reduce mineral phosphate fertilizer supplementation and promote plant growth. Nevertheless, only several P-solubilizing microorganisms capable of solubilizing both organic and mineral sources of soil phosphorus have been identified up to now. The aim of this study was to evaluate the inorganic soil phosphate solubilizing activity of phytate-hydrolyzing *Pantoea brenneri* soil isolates. We showed that the strains efficiently solubilize a variety of inorganic phosphates. We optimized the media composition and culturing conditions to improve the solubilization efficiency of the strains and investigated the mechanisms of their phosphate solubilization. Through HPLC analysis, it was determined that *P. brenneri* produce oxalic, malic, formic, malonic, lactic, maleic, acetic, and citric acids as well as acid and alkaline phosphatases while growing on insoluble phosphate sources. Finally, we analyzed the influence of *P. brenneri* strains with multiple PGP-treats on plant growth in greenhouse experiments and showed their ability to promote growth of potato.

## 1. Introduction

Traditional agriculture plays a leading role in food supply for Earth’s growing population. The latest projections by the United Nations indicate that the global population could increase to 9.7 billion by 2050 and reach 10.4 billion by 2100 [1]. World population growth is expected to pose significant challenges to food security, and to address these challenges, strategies in promoting sustainable agriculture should be proposed. Despite the great improvements achieved by the use of mineral fertilizers and chemical pesticides in agriculture, the environmental problems associated with them are becoming increasingly apparent. Therefore, it is of great interest to develop alternative technologies in agricultural production that allow preserving and increasing the productivity of cultivated crops.

Phosphorus (P) is one of the most important macronutrients in plants and is essential for their growth and development. The P content reaches 0.2% of the plant dry weight [2]. However, despite high concentrations of total P in soil, the concentration of bioavailable phosphorus rarely exceeds 10 μM [3], which significantly reduces the agronomic efficiency of mineral P-fertilizers [4]. Therefore, development of approaches to increase the bioavailability of phosphorus in soil is one of the main objectives in modern agriculture. An environmentally safe and promising method is the targeted use of soil rhizosphere microorganisms as biofertilizers. Species of the genera *Acinetobacter*, *Azospirillum*, *Bacillus*, *Burkholderia*, *Enterobacter*, *Klebsiella*, *Ochrobactrum*, *Pantoea*, *Paenibacillus*, *Pseudomonas*, *Rhizobium*, *Serratia*, and *Sphingomonas* have been shown to improve plant productivity and health through various mechanisms, including phosphate solubilization and mineralization [5,6,7]. The application of such phosphate solubilizing microorganisms (PSMs) in soil has been shown to reduce mineral phosphate fertilizer supplementation by 50% while maintaining crop yield.

Approximately 30–65% of total soil phosphorus is present in organic forms, while the remaining 35–70% is present in inorganic forms [8]. Inorganic phosphorus in soil is categorized into plant-available, sorbed, and mineral phosphorus. Plant-available phosphorus consists of inorganic phosphates dissolved in water/soil solution and accessible for plant metabolism. Sorbed phosphorus is comprised of inorganic phosphates attached to clay surfaces, iron (Fe), aluminum (Al), and calcium (Ca) oxides in soil. The release of phosphorus from this pool is gradual and slow. Mineral phosphorus consists of phosphorus minerals such as apatite, rock-phosphate, strengite, phosphorite, and variscite. Phosphorus release from this pool happens only when the mineral weathers and dissolves in soil water [9].

In agricultural soils, organic P is mainly found in the form of inositol hexakisphosphate (IP6; phytate). Soil phytate can be produced through the transformation of soil inorganic P by microorganisms, from plant tissues, and in monogastric animal manures [10]. However, soil phytates cannot be directly utilized by plants. Phosphate ester (C-O-P), phosphoanhydride (P-O-P), or phosphonate (C-P) must first be dephosphorylated through phytase-mediated hydrolysis [11]. Plants and microbes have developed strategies to improve phytate bioavailability by secreting organic acids and hydrolyzing enzymes—namely, phytases [10]. Although worldwide applications of phytase are mainly, if not solely, concentrated in animal nutrition as feed additives in grain-based diets of swine, poultry and fish [12], nowadays, there is a growing interest in their application in agriculture.

Phosphate-solubilizing bacteria (PSB) are generally associated with the plant rhizosphere and known to enhance bioavailability of soil P. Nevertheless, only several PSB capable of solubilizing both organic and mineral sources of phosphorus have been identified up to now [13,14]. To address the lack of phosphorus in agricultural soils, it is essential to consider the application of microorganisms capable of solubilizing both organic and inorganic forms of soil P.

In previous studies, our research group isolated and characterized the phytate-hydrolyzing strains from the soils of the Republic of Tatarstan, identified by MLSA-analysis as *P. brenneri*. We showed that the ability of *P. brenneri* strains to hydrolyze soil organic compounds, phytates, is associated with glucose-1-phosphatase enzyme that possesses phytase activity [15,16]. Moreover, multiple plant-growth-promoting (PGP) properties of these strains have been reported, namely, the secretion of hydrolytic enzymes (phytase, protease, cellulase), cyanides (HCN), and the production of auxin and siderophores. Additionally, *P. brenneri* strains exhibit antifungal activity against phytopathogenic *Fusarium* [17]. The objectives of this study were to understand the mechanisms of inorganic phosphate solubilization during organic acid secretion and phosphatase production of four rhizosphere *P. brenneri* isolates and to evaluate their potential for increasing potato yield in greenhouse trials.

In the present study, we evaluated the inorganic soil phosphates solubilizing activity of phytate-hydrolyzing *P. brenneri* strains. We optimized the media composition and culturing conditions to improve the solubilization efficiency of the strains and investigated the mechanisms of their phosphate solubilization, including genomic insight. Finally, we analyzed the influence of *P. brenneri* strains on plant growth in greenhouse experiments.

## 2. Materials and Methods

### 2.1. Bacterial Strains and Culture Media

*P. brenneri* strains 3.1, 3.2, 3.5.2, and 3.6.1 were originally isolated from soil samples of the Republic of Tatarstan according to their phytate-hydrolyzing activity [11]. The strains were cultured on LB medium containing 10 g tryptone, 5 g yeast extract, and 5 g NaCl per 1 L distilled water, at pH 7.0. The agar medium (LA) included an additional 2% agar.

P-solubilization ability was evaluated using the National Botanical Research Institute’s Phosphate growth medium (NBRIP): glucose 10 g, MgCl_2_ × 6H_2_O 5 g, MgSO_4_ × 7H_2_O 0.25 g, KCl 0.2 g, (NH_4_)_2_SO_4_ 0.1 g, distilled water 1 L; the pH was adjusted to 7.0 ± 0.2 before sterilization [18]. Tricalcium phosphate (Ca_3_(PO_4_)_2_), calcium hydroorthophosphate (CaHPO_4_), hydroxyapatite, phosphorite, aluminum phosphate (AlPO_4_), and iron phosphate (Fe_3_(PO_4_)_2_) at a concentration of 5 g/L each were used as single phosphorus sources. Solid NBRIP medium contained an additional 1.5% agar. The bacterial strains were spot inoculated on agar plates and incubated at 30 °C for 5–10 days.

### 2.2. Determination of the P-Solubilization Ability of Bacteria

To determine the P-solubilization ability, strains were screened for clear phosphate-solubilizing zones around the colonies after 5–10 days of incubation at 30 °C on the agar plates with NBRIP medium. NBRIP agar plates were supplemented with one of the insoluble phosphate sources: Ca_3_(PO_4_)_2_, CaHPO_4_, hydroxyapatite, phosphorite, AlPO_4_, or Fe_3_(PO_4_)_2_ at a concentration of 5 g/L each. The diameter of the bacterial colony and the halo zone were measured on the 4th day and then were used to evaluate the Phosphate Solubilization Index (PSI) as the ratio of the total diameter (colony + halo zone) to the colony diameter [19]. Each measurement was repeated in triplicate and the average values were reported.

To quantify P-solubilization, the level of free phosphates and pH values of the culture medium were measured, and the efficiency of P-solubilization was calculated. *P. brenneri* strains were cultured in liquid NBRIP medium with various insoluble P sources. The selected isolates were initially grown in LB broth, and then cells were pelleted by centrifugation at 6000 rpm for 10 min. The cell pellets were washed twice with NBRIP medium without phosphate. The washed cells were added to a 100 mL conical flask containing 20 mL of NBRIP medium with a final OD_600_ of 0.1. The flasks were incubated at 28–30 °C and 200 rpm for 3–7 days. A non-inoculated medium was used as a blank control. Cells were then pelleted by centrifugation at 6000 rpm for 10 min. A cell-free supernatant was used for further analysis. The pH of the bacterial suspension was assayed by reading on a pH meter. The liberated inorganic phosphates were measured by a modification of the ammonium molybdate method [20]. The reaction mixture contained 100 μL of cell-free supernatant and 750 μL of freshly prepared 2:1:1 AAM solution (acetone—5 N H_2_SO_4_—10 mM ammonium molybdate). After incubation for 2 min, 50 μL of 1M citric acid was added to the reaction mixture. Optical density was measured at 355 nm on a 2550 Microplate Reader (Bio-Rad, Hercules, CA, USA). The concentration of solubilized phosphates in the liquid medium was calculated using the equation of potassium dihydrogen phosphate (KH_2_PO_4_) obtained from the calibration curve. The efficiency of P-solubilization was calculated by dividing the amount of free phosphates in the experimental flasks by the amount in the abiotic control and then multiplying the result by 100%. Each measurement was repeated in triplicate and the average values were reported.

### 2.3. Optimization of Culture Conditions and Media Composition for P-Solubilization

To assess the effect of various carbon and nitrogen sources in the medium on the P-solubilization efficiency, *P. brenneri* strains were grown in liquid NBRIP medium with 5 g/L of tricalcium phosphate containing alternative types of carbon and nitrogen sources at 30 °C and pH 7.0 on a rotary shaker at 200 rpm. Glucose, xylose, sucrose, maltose, galactose, fructose, and mannitol were used as carbon sources (at a concentration of 10 g/L), whereas ammonium sulfate ((NH_4_)_2_SO_4_), potassium nitrate (KNO_3_), yeast extract, peptone, and urea were adopted as sources of nitrogen (at a concentration of 0.1 g/L).

To investigate the influence of initial pH, temperature, and rotation speed on P-solubilization, *P. brenneri* strains were cultured in the liquid NBRIP medium with 5 g/L of tricalcium phosphate. The cultivation was performed at initial pH of 7.0 and different temperatures of 25 °C, 30 °C, 37 °C, and 42 °C on a rotary shaker at 200 rpm; or at 30 °C and different initial pH of 2.0, 4.0, 6.0, 7.0, and 9.0 on a rotary shaker at 200 rpm; or at 30 °C and initial pH 7.0 on a rotary shaker at different rotation speeds of 100, 170, 200, and 230 rpm. Liberated phosphates in the culture media were determined on the 3rd day of cultivation following the above method. Each measurement was repeated in triplicate and the average values were reported.

### 2.4. Determination of Produced Organic Acids

The organic acid profile of *P. brenneri* strains was analyzed through HPLC [21]. All four strains were inoculated into a 100 mL flask with 50 mL of liquid NBRIP medium, containing tricalcium phosphate, phosphorite, or hydroxyapatite as the sole source of phosphorus. The flasks were incubated on a shaker at 30 °C and 200 rpm for 7 days. Cells were pelleted by centrifugation at 10,000 rpm for 20 min. The obtained supernatant was filtered with a 0.22 μm membrane filter (MilliporeSigma, Burlington, VT, USA) and then loaded in chromatographic columns. Samples (20 μL) were analyzed using a Knauer Smartline HPLC system (Knauer, Berlin, Germany) using reverse-phase chromatography. Organic acids were separated on Spherisorb ODS2 C-18 column (4.6 × 250 mm, 5 μm) (Waters, Milford, CT, USA) at 35 °C. Elution was carried out in isocratic mode with a flow rate of 1 mL min-1. The mobile phase used in this case consisted of 25 mM K_2_HPO_4_ (Carl Roth, Karlsruhe, Germany), with a pH of 2.1. Signals were detected at 200, 205, and 210 nm and compared with Organic Acid Analysis Standard (Bio-Rad, Hercules, CA, USA). In addition to the commercial standard kit, malonic (22 mM), lactic (129 mM), maleic (19 mM), tartaric (18 mM), and pyruvic acid (23 mM) (Gradient grade) were used as controls. Peak area and retention time compared with those of standards were used for the quantification of organic acids. The values were presented as the mean of three replicates.

### 2.5. Alkaline and Acidic Phosphatase Activity Detection

Phosphatase activity was determined according to the modified method of Magallon-Servin et al. [22]. The activity of acid and alkaline phosphatases was determined by the hydrolysis of the substrate para-nitrophenyl phosphate (pNPP) at their optimal pH values of 6.5 and 11.0, respectively [23]. *P. brenneri* strains were grown at 30 °C for 7 days on liquid NBRIP media with tricalcium phosphate, phosphorite, or hydroxyapatite as the sole source of phosphorus. On the 1st, 3rd, 5th, and 7th days of cultivation, cell-free culture media supernatants were collected and used for phosphatase assay. A non-inoculated medium served as the negative control. The reaction mixture contained 200 μL modified universal buffer (MUB), 50 μL 0.115 M pNPP (Merck KGaA, Darmstadt, Germany) in MUB and 10–50 μL of culture media supernatants. The reaction was carried out at 37 °C for 30 min and terminated by adding 200 μL of 0.5 M NaOH and 50 μL of 0.5 M CaCl_2_. Optical density was measured at 420 nm on a 2550 Microplate Reader (Bio-Rad, Hercules, CA, USA). One unit (U) of phosphatase activity was defined as the amount of enzyme required to liberate 1 μmol of inorganic phosphorous from pNPP per min at 37 °C.

### 2.6. Genomic Screening for the Genes Involved in Phosphate Metabolism

The genome of *P. brenneri* strain 3.5.1 has been previously sequenced and assembled and is accessible in the NCBI GenBank as JMRT00000000.2 (https://www.ncbi.nlm.nih.gov/nuccore/JMRT00000000, accessed on 15 April 2015). Genome annotation was performed with RAST (https://rast.nmpdr.org/) accessed on 13 April 2015. Annotated and predicted gene sequences were evaluated to screen the specific pathways of phosphorus metabolism in bacteria. The ascribed gene functions were manually analyzed and compared with the relevant pathways available in the Kyoto Encyclopedia of Genes and Genomes (KEGG) pathway databases (https://www.genome.jp/kegg/pathway.html, accessed on 13 April 2015).

### 2.7. Seed Germination Assay

Wheat seeds (Zlata and Tulaikovskaya cultivars) were surface sterilized in 70% ethanol for 1 min, then treated for 60 s with a 2% NaOCl solution, and rinsed 5 times with sterile distilled water. The inoculum for *P. brenneri* strain 3.5.2 was prepared from the culture grown in LB medium at 30 °C for 24 h. Cells were harvested by centrifugation at 10,000 rpm for 10 min, resuspended in sterile 1% sucrose solution (final OD_600_ 0.1), and used for seed inoculation. Uninoculated seeds treated with a sterile 1% sucrose solution were used as a negative control. The treatment was carried out using the following experimental design: six Petri dishes with 20 seeds on each were used in the experimental group, inoculated with *P. brenneri* strain 3.5.2; the same quantity was used in the control group, treated with 1% sucrose solution. The bacterized seeds were germinated in Petri dishes on moist filter paper, sealed with parafilm, and kept at 25–27 °C. The germination index was analyzed on the 7th day, and the root and shoot length were measured on days 3, 7, and 11. The relative seed germination was expressed as a percentage of the number of germinated seeds [24]. Germinated seeds were considered to have at least two roots larger than the length of the seed and a sprout of at least half of its length. The values were presented as the mean of three replicates.

### 2.8. Greenhouse Experiments

The bacterial strains were grown in the LB medium at 28 °C and 200 rpm until optical density (OD_590_) reached 1.0. After growth, cells were harvested by centrifugation at 10,000 rpm for 10 min and resuspended in sterile tap water. The bacterial suspension of *P. brenneri* strain 3.1 with a concentration of 1.9 × 10^8^ CFU/mL, *P. brenneri* strain 3.2 with a concentration of 2.3 × 10^8^ CFU/mL, and *P. brenneri* 3.5.2 with a concentration of 2 × 10^8^ CFU/mL were prepared for the test.

To evaluate plant growth promotion activities of the *P. brenneri* strains on potato plants (*Solanum tuberosum* L.), medium-size tubers of the cultivar Desiree were used. Potato tubers were obtained from the Collective Use Center “Bioresource Potato Collection” (https://www.ckp-rf.ru/, Reg. No. 471948, 9 January 2017), partially supported within the framework of the State Assignment of the Tatar Research Institute of Agriculture (TRIA FRC KazSC RAS). Greenhouse trials were carried out at the experimental base of TRIA FRC KazSC RAS with the following experimental design. There were two treatment options for each bacterial suspension: (1) pre-planting treatment of potato tubers (27 tubers), and (2) pre-planting treatment along with double irrigation of the soil in the root zone of potato plants with bacterial suspensions immediately after planting and in one month (27 tubers). Sterile tap water was used as an uninoculated control treatment. Single tubers were dipped in the inoculum for 15 min and then placed in 2 L pots containing high-moor peat from the deposit of OJSC “Paranginskoe peat enterprise” (41.36% ash, 41.36% organic matter, 633 mg/kg alkaline hydrolysable nitrogen, 400 mg/kg P_2_O_5_, 260 mg/kg K_2_O, 71.3 meq/100 g hydrolytic acidity, pH 5.1). A week before planting, mineral fertilizer (N88P94K96) was added to the potting soil. The pots were filled and watered up to full soil moisture capacity. One tuber was planted in each pot. Soil moisture was maintained at the required level with weekly watering. Plants were harvested after 3 months. The productivity and development of the potato plants were assessed based on the following parameters: plant height; the number of stems; the presence and number of yellow leaves; the presence and number of flowers; and the number and weight of tubers.

### 2.9. Statistical Analysis

All analyses were performed on three biological replicates. The obtained data were processed using Statgraphics Plus 5.0. and GraphPad Prism 7.05 statistical software, and are presented as the mean ± standard deviation (SD). Student’s *t*-test analysis, analysis of variance (ANOVA) [25], and Tukey’s test were used to calculate the data variance with *p* < 0.05 representing a significant difference. Data on studied plant parameters of the greenhouse experiments were analyzed statistically by ANOVA using R 4.2.2 software [26].

## 3. Results

### 3.1. P-Solubilizing Ability of P. brenneri Strains on Different Insoluble Phosphorus Sources

#### 3.1.1. Phosphate Solubilization Ability on Solid Media

**Figure 1:**
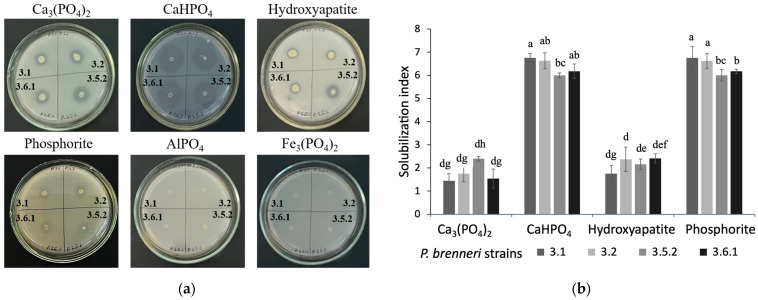
P-solubilization activity of *P. brenneri* strains on NBRIP agar plates with different P-sources. (**a**)—Halo zone formation. (**b**)—P-solubilization indexes (PSI) of the bacterial isolates. Data are shown as Mean ± SD. Means followed by different letters indicates significant difference (*p* < 0.05) according to ANOVA—Tukey’s test.

We started with evaluating the ability of *P. brenneri* strains to solubilize P from different insoluble inorganic P-sources on NBRIP agar plates. Tricalcium phosphate (Ca_3_(PO_4_)_2_), calcium hydroorthophosphate (CaHPO_4_), hydroxyapatite, phosphorite, aluminium phosphate (AlPO_4_), or iron phosphate (Fe_3_(PO_4_)_2_ were added as the sole source of phosphorus. After 7 days of incubation, all four studied strains showed well-developed halo zones on plates containing Ca_3_(PO_4_)_2_, CaHPO_4_, hydroxyapatite, and phosphorite (Figure 1a). The strains produced cylindrical, pale yellow, translucent, and glossy colonies but did not form halo zones on plates containing AlPO_4_ and Fe_3_(PO_4_)_2_ (Figure 1a). On NBRIP agar plates with Ca_3_(PO_4_)_2_, *P. brenneri* 3.1, and *P. brenneri* 3.2 formed semicylindrical colonies that were yellow, translucent, and glossy; *P. brenneri* 3.5.2 and *P. brenneri* 3.6.1 formed cylindrical colonies that were yellow, opaque, and glossy. The P-solubilizing index (PSI) was higher for all four strains on NBRIP with CaHPO_4_ and phosphorite (6.39 ± 0.4), as shown in Figure 1b. The PSI for all strains did not exceed 2 on media containing hydroxyapatite and Ca_3_(PO_4_)_2_. Bioassays on solid media indicated that the studied *P. brenneri* strains can mobilize phosphate from various inorganic sources.

#### 3.1.2. Phosphate Solubilization Efficiency on Liquid Media

**Figure 2:**
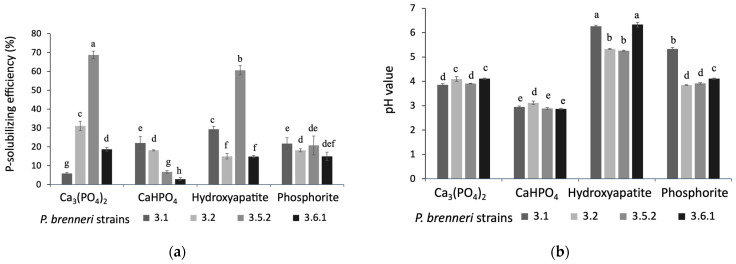
P-solubilization capability of *P. brenneri* strains on liquid NBRIP medium with different P sources. (**a**)—P-solubilization rates, (**b**)—pH-values; the initial pH of uninoculated medium was 7.0. Data are shown as Mean ± SD. Means followed by different letters indicates significant difference (*p* < 0.05) according to ANOVA—Tukey’s test.

In order to screen for the strain with the maximum P-solubilizing efficiency in different P-sources, we inoculated the isolates into liquid NBRIP media supplemented with either tricalcium phosphate (Ca_3_(PO_4_)_2_), calcium hydroorthophosphate (CaHPO_4_), hydroxyapatite, or phosphorite. For quantitative estimation of phosphate solubilizing activity, the level of free phosphates accumulated during bacterial incubation and the pH value of the cultivation medium were measured (Figure 2). Among the four strains tested, *P. brenneri* 3.5.2 demonstrated significantly (*p* < 0.01) high P-solubilizing efficiency on Ca_3_(PO_4_)_2_, reaching 68.74%, and on hydroxyapatite, reaching 61.22% (Figure 2a). On NBRIP media containing phosphorite and CaHPO_4_, the P-solubilizing efficiency was less than 22% for all strains.

During the cultivation on NBRIP media with different sources of inorganic phosphorus, all bacteria decreased the pH values of the medium, starting from an initial pH of 7.0. While there was no significant difference in pH levels between the strains on Ca_3_(PO_4_)_2_ and CaHPO_4_, *P. brenneri* strain 3.5.2 displayed a significantly (*p* < 0.05) lower pH value (5.25) compared to strains 3.1 and 3.6.1 on the medium with hydroxyapatite and a significantly (*p* < 0.05) lower pH value (3.92) compared to strain 3.1 on the medium with phosphorite (Figure 2b). Based on the statistical analysis, *P. brenneri* strain 3.5.2 was selected for the following experiments.

**Figure 3:**
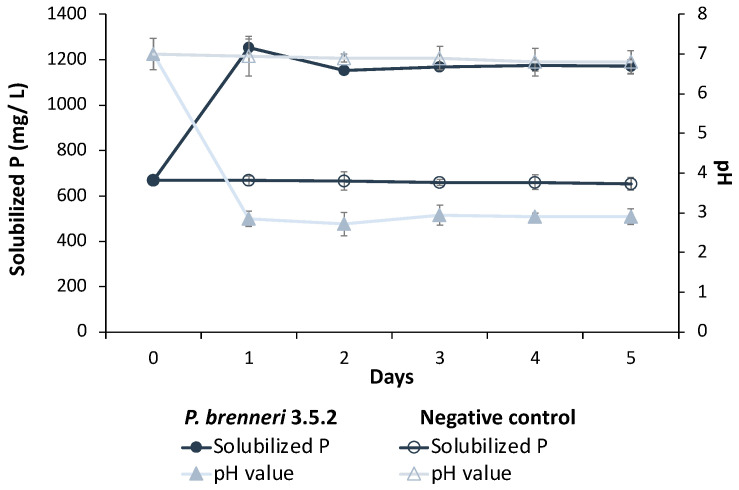
Dynamics of P-solubilization rates and pH values on liquid NBRIP medium with insoluble tricalcium phosphate as a sole source of phosphorus. Data are shown as Mean ± SD.

To understand the dynamics of phosphorus release from Ca_3_(PO_4_)_2_ by *P. brenneri* strain 3.5.2, we measured the number of liberated phosphates and pH values of the NBRIP medium every 24 h for five days of incubation. The concentration of free phosphates reached a peak of 1253.49 mg/L with an 87% solubilization efficiency within 24 h of inoculation and remained stable until the end of the five-day incubation period (1170.21 mg/L by the 5^th^ day of incubation) (Figure 3). The pH level of the medium decreased from 7.0 to 2.85 immediately after 24 h of cultivation and remained constant throughout the experiment (Figure 3). Therefore, the plateau level of free phosphates observed in the first 24 h was correlated with the pH changes.

### 3.2. Optimization of Culture Conditions and Media Composition for P-Solubilization

**Figure 4:**
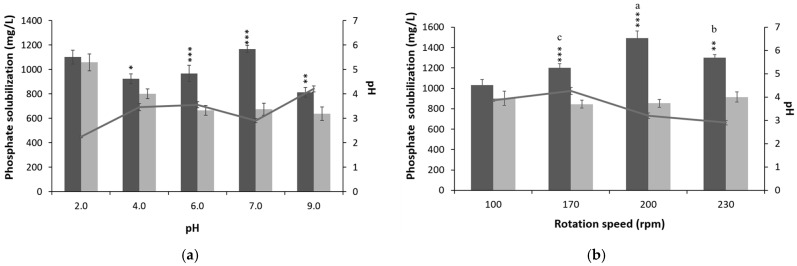

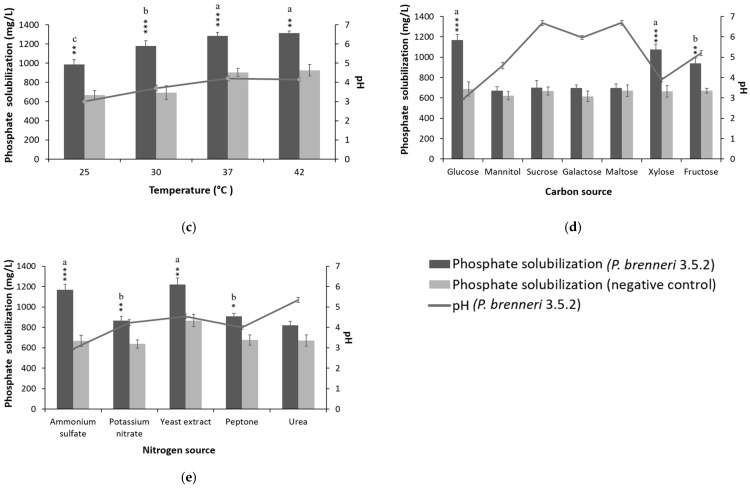
P-solubilization rates and change in pH of medium during P-solubilization of *P. brenneri* 3.5.2 for different pH values (**a**), rotation speed (**b**), temperatures (**c**), carbon sources (**d**), and nitrogen sources (**e**). Data are shown as Mean ± SD. * *p* < 0.05, ** *p* < 0.01, and *** *p* < 0.001 vs. Negative control group. Means followed by different letters indicates significant difference (*p* < 0.05) according to ANOVA—Tukey’s test.

Next, we assessed the effect of cultivation conditions and microelement sources on phosphate mobilization by *P. brenneri* 3.5.2 (Figure 4). The initial pH of the cultivation medium, rotation speed, and temperature of cultivation had a valuable effect on the solubilization ability of Ca_3_(PO_4_)_2_ by bacteria. *P. brenneri* 3.5.2 showed significantly high (*p* < 0.001) amounts of soluble P (1167 mg/L) and solubilization efficiency (75%) when the pH was set at 7.0 (Figure 4a). Additionally, a significant decrease in pH was observed, dropping from 7.0 to 2.9. While high concentrations of soluble P (1103 mg/L) were also observed at the initial pH of 2.0, these values did not differ significantly from the sterile control, suggesting that the release of phosphates may be due to the initially low pH of the medium rather than bacterial activity.

*P. brenneri* 3.5.2 exhibited significantly (*p* < 0.001) high P solubilization (1494 mg/L of free phosphates) at a rotation speed of 200 rpm (Figure 4b). In this case, the maximum acidification of the medium was also observed: pH decreased from 7.0 to 3.1. Next, we determined the impact of temperature on the P-dissolving rate of *P. brenneri* 3.5.2 (Figure 4c). The highest amount of free phosphates was observed at 37 °C and 42 °C, reaching about 1200 mg/L. However, when compared to the uninoculated control, the overall P-solubilizing efficiency was higher at 30 °C (70.01% efficiency), with a reduction in pH value of the medium by 3.6.

Furthermore, we investigated the impact of various carbon and nitrogen sources on the phosphate-solubilizing efficiency of *P. brenneri* 3.5.2 (Figure 4d). When glucose, mannitol, sucrose, galactose, maltose, xylose, or fructose was used as the carbon source, the decrease in pH of the medium was by 4.1, 2.49, 0.32, 1.02, 0.31, 3.11, or 1.8, respectively. Significantly (*p* < 0.001) high P-solubilization—1168.03 mg/L and 1115.24 mg/L—was observed when glucose and xylose were used as the carbon source. Interestingly, when fructose was used as the carbon source, there was only a slight decrease in pH during inoculation (from 7.0 to 5.2) together with P-solubilization efficiency of 40.81%. However, sucrose and maltose did not significantly increase the number of free phosphates or decrease the pH level compared to uninoculated media, indicating that these sugars cannot be utilized as carbon sources by *P. brenneri* 3.5.2.

In terms of nitrogen sources, ammonium sulfate and yeast extract resulted in a significantly (*p* < 0.001) large number of liberated phosphates—1191.05 mg/L and 1219.95 mg/L, respectively (Figure 4e). However, a high number of free phosphates were also observed in the uninoculated flask with yeast extract, resulting in a total P-solubilization efficiency not exceeding 40%. Compared to negative control, ammonium sulfate was found to be the most effective, with a P-solubilization efficiency of 74.56% and a significant decrease in pH from 7.0 to 2.94. The use of potassium nitrate (KNO_3_) and peptone as nitrogen sources did not result in P-solubilization efficiencies above 37%. Lastly, the utilization of urea as a nitrogen source did not result in a significant difference in the number of free phosphates between the experimental and control flasks.

Thus, taken together, for optimal P-solubilization efficiency of tricalcium phosphate by *P. brenneri* 3.5.2, we recommend culturing the bacteria on a liquid medium with a pH of 7.0 and containing glucose and ammonium sulfate as the carbon and nitrogen sources, respectively, at 30 °C on a rotary shaker at 200 rpm.

### 3.3. Determination of Produced Organic Acids by HPLC

In order to determine the composition and concentration of organic acids produced by *P. brenneri* strains, we performed an HPLC analysis. The *P. brenneri* strains 3.2 and 3.5.2 were cultured on liquid NBRIP media containing phosphorite, hydroxyapatite, or tricalcium phosphate as the sole source of phosphorus. We detected several types of organic acids in different concentrations in culture media supernatant of bacteria. *P. brenneri* strains produced oxalic, malic, formic, malonic, lactic, maleic, acetic, and citric acids (Table 1, Figure S1). Malic and formic acids were found to be the most abundant (>10 mM). Interestingly, the type and concentration of organic acids varied depending on the phosphorus source in the medium. The highest concentration of formic acid was observed when both strains were grown on the Ca_3_(PO_4_)_2_-containing medium—specifically, 11.2 mM for *P. brenneri* 3.2 and 10.1 mM for *P. brenneri* 3.5.2—but oxalic acid was not produced under this condition by either strain.

Furthermore, differences in the biosynthesis of organic acids were also observed between the strains (Table 1). For instance, *P. brenneri* 3.2 only produced malonic acid in the presence of hydroxyapatite in the medium and did not secrete lactic, acetic, and citric acids while growing on this phosphorus source. *P. brenneri* 3.5.2 did not produce lactic acid on the medium with phosphorite but did produce it when cultivated on hydroxyapatite and tricalcium phosphate. The production of malic acid also differed among the strains: the highest concentration was observed during growth on tricalcium phosphate for strain 3.2 (12.8 mM) and on hydroxyapatite for strain 3.5.2 (13.8 mM).

### 3.4. Alkaline and Acidic Phosphatase Activity of P. brenneri Strains

Both alkaline phosphatase (ALP) and acid phosphatase (ACP) were secreted by *P. brenneri* strains during growth on NBRIP containing hydroxyapatite, phosphorite, or tricalcium phosphate. The maximum activity of ALP was demonstrated by *P. brenneri* strain 3.2 (4.85 U/100 mL) on the 7th day of incubation on the medium with hydroxyapatite. The maximum activity of ACP was demonstrated by *P. brenneri* strain 3.5.2 (2.149 U/100 mL) on the 5th day of incubation on the medium containing hydroxyapatite.

### 3.5. Genes Involved in Phosphate Metabolism of P. brenneri

Previously, it has been shown that *P. brenneri* strains possess multiple PGP-traits such as Indole acetic acid (IAA) and siderophore production, nitrogen fixation, and antifungal activity against phytopathogenic micromyces [17]. The genome of *P. brenneri* strain 3.5.1 was annotated with RAST and screened for the genes involved in PGP-features [27]. Besides PGP-associated genes, the ones involved in phosphate metabolism were identified (Table 2). The *P. brenneri* strain 3.5.1 genome contains glucose dehydrogenase gene (*gcd*) and its cofactor pyrroloquinoline quinone (*pqq*) operon (Table 2), which catalyze the production of gluconic acid—one of the organic acids involved in phosphate solubilization. The sequence analysis of *gcd* showed its maximum sequence homology with *gcd* of *P. agglomerans* (93.68%) and *P. conspicua* (92.63%). The sequence analysis of *pqqE* showed its sequence homology with *pqqE* of *P. conspicua* (98.42%), *P. agglomerans* (98.16%), and *P. vagans* (96.84%).

Moreover, we identified the cluster of *phn*-genes responsible for C-P lyase activity and utilization and transport of phosphonates, which suggest the ability of the strain to degrade phosphonates. Moreover, genes of exopolyphosphatase (*ppx*) and polyphosphate kinase (*ppk*) biosynthesis are present in the genomes of *P. brenneri*. These genes are responsible for the ability of the strain to mobilize inorganic polyphosphates (polyP), which can also be served as source of inorganic phosphate.

A full cluster of phosphate-specific transporters (PstSCAB) was identified in the genome of bacteria. *P. brenneri* has two phosphate-starvation inducible (*psr*) genes, *psiE* and *psiF*. Finally, we discovered genes-encoding enzymes responsible for organic phosphate mineralization in *P. brenneri* genome: 3-phytase, glucose-1-phosphatase with phytase activity, and alkaline phosphatase (Table 2).

### 3.6. Effect of P. brenneri on Wheat Seed Germination

Next, we assessed the impact of *P. brenneri* strain 3.5.2 on the germination and growth of two wheat (*Triticum aestivum*) cultivars, Zlata and Tulaikovskaya. Seeds of both cultivars began to germinate after 1 day of incubation. By day 7, *P. brenneri* strain 3.5.2 had significantly increased seed germination rates by 97% for cv. Zlata and 96% for cv. Tulaikovskaya, compared to control groups which had rates of 89% and 86%, respectively.

**Figure 5:**
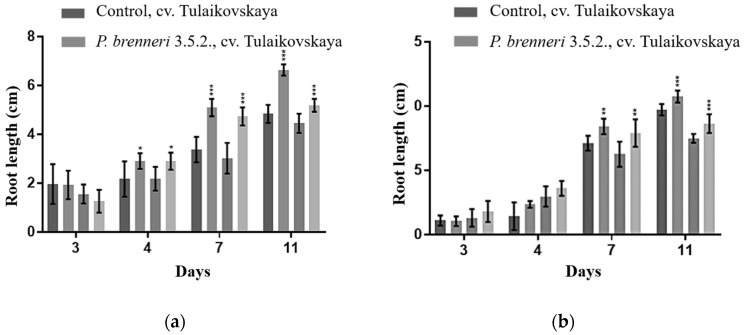
Seedling root (**a**) and shoot (**b**) lengths of wheat (cv. Tulaikovska and cv. Zlata) treated with *P. brenneri* strain 3.5.2 for different durations. Data are shown as Mean ± SD. * *p* < 0.05, ** *p* < 0.01, and *** *p* < 0.001 vs. Negative control group.

Further analysis on day 11 revealed that *P. brenneri* 3.5.2 also stimulated root and shoot growth of the seedlings (Figure 5). The root length of cv. Tulaikovskaya seeds inoculated with *P. brenneri* 3.5.2 was increased by 37.2% compared to the control, and that of cv. Zlata showed a 16.6% increase. The shoot length of cv. Tulaikovskaya seeds inoculated with *P. brenneri* 3.5.2 was increased by 10.7% compared to the control, while that of cv. Zlata showed a 15.4% increase. The obtained results indicate that bacterial inoculation had a positive impact on the growth of both wheat cultivars, particularly, in stimulating root and shoot growth in wheat seedlings.

### 3.7. Inoculation Effects on Potato Traits in Greenhouse Experiments

The following parameters of plant morphology and productivity were assessed: the height and number of stems, the presence and number of yellow leaves and flowers, the number and weight of tubers, and tuber yield. Statistical analysis showed no significant difference between the studied traits and the treatment options of bacterial suspensions. Accordingly, an additional application of bacterial suspensions to the soil after pre-planting treatment was not necessary.

**Figure 6:**
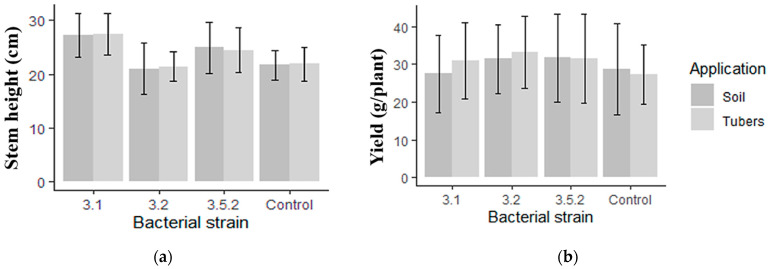
Effect of bacterial inoculation on stem height (**a**) and productivity (**b**) of potato (cultivar Desire) grown in pots in greenhouse conditions. Data are shown as Mean ± SD. Comparison between treatments was carried out by one-way analysis of variance (ANOVA).

On the whole, inoculation with bacteria yielded positive effects on plant growth parameters. It was shown that inoculation of potato plants with *P. brenneri* strains did not have a statistically significant effect on the presence and number of yellow leaves and flowers, or the number and weight of tubers. Inoculation with *P. brenneri* strains 3.1 and 3.5.2 showed maximum increases in stem height by 26% and 14%, respectively (Figure 6a). Along with this, treatment with *P. brenneri* strain 3.5.2 significantly reduced the number of stems compared to the control, whereas inoculation with *P. brenneri* 3.2 did not affect stem height and quantity. Treatment with *P. brenneri* strains 3.1, 3.2, and 3.5.2 had a positive effect on the yield of potato plants (Figure 6b). Specifically, the tuber yield of potato plants when treated with *P. brenneri* strain 3.2 was 33.2 g/plant versus 28 g/plant for uninoculated control plants.

## 4. Discussion

Phosphorus is one of the least available macronutrients for plants in soil [21]. A large amount of phosphorus from chemical fertilizers rapidly becomes immobilized due to its fixation, adsorption, and precipitation. Phosphate solubilizing bacteria (PSB) are capable of hydrolyzing organic and inorganic phosphorus into soluble forms, thus making it bioavailable to plants [28]. In this study, we determined the phosphate-mobilizing activity of *P. brenneri* strains and evaluated the effect of bacterial inoculation on potato tubers. Previously, we isolated phytate-hydrolyzing *P. brenneri* strains from the soil samples of the Republic of Tatarstan. We established biochemical heterogeneity within the *P. brenneri* species [16], which underlines the importance of studying various strains of this species.

To start with, we investigated the ability of *P. brenneri* strains 3.1, 3.2, 3.5.2, and 3.6.1 to solubilize phosphorus from different insoluble inorganic P-sources. In our analysis, we used two distinct types of soil inorganic P forms. The first was sorbed phosphorus, which consisted of iron (Fe), aluminum (Al), and calcium (Ca) oxides. The second was mineral phosphorus, which was comprised of primary phosphate minerals such as apatite and phosphorite [9]. All isolates were capable of solubilizing Ca_3_(PO_4_)_2_, CaHPO_4_, hydroxyapatite, and phosphorite. The absence of halo zones on Fe–P- and Al–P-containing agar plates suggests that *P. brenneri* strains are more suitable for application in alkaline soil. However, further evaluation of phosphate-solubilizing activity on liquid medium indicated that visible halo zone formation and PSI cannot serve as a reliable criterion for P-solubilizing bacteria screening. This was mentioned by other authors as well—no significant positive correlation was found between P solubilization in liquid media and the halo zone produced in solid media by *A. xylosoxidans* PSB5 and two *Bacillus* strains studied by Ibáñez et al. [29]. Halo zones production on solid medium may be used for screening of PSM, but not in quantitative estimation of their P-solubilization efficiency [30]. Thus, some strains, in particular, *P. brenneri* 3.5.2, on agar media with Ca_3_(PO_4_)_2_ and hydroxyapatite exhibited low PSI but the highest phosphate solubilizing activity (1341.66 и 1190.66 mg/L) on liquid medium. The study by Chen and Liu [31] reported that *Pantoea* sp. S32 also showed the highest phosphate releasing capacity on the medium with Ca_3_(PO_4_)_2_ with the number of free phosphates amounted to 1256.67 mg/L. The dynamics of phosphorus release from Ca_3_(PO_4_)_2_ by *P. brenneri* strain 3.5.2 was studied for five days. The results showed that the concentration of free phosphates peaked on the first day and remained relatively stable over the following days of experiment. Similar results were reported by Walpola et al. [32]: it was shown that *P. agglomerans* and *P. rodasii* are capable of utilizing phosphorus from the NBRIP medium with Ca_3_(PO_4_)_2_ and phosphate solubilization occurred mainly on days 1–2 of incubation (810 µg/mL) and on days 2–3 (788 µg/mL), respectively.

As heterotrophic bacteria, phosphate solubilizers require carbon and energy sources. In the present study, by using glucose, xylose, fructose, mannitol, galactose, sucrose, or maltose as the carbon source, the P solubilization efficiency of *P. brenneri* 3.5.2 amounted to 74.56, 62.74, 40.81, 8.05, 12.6, 4.78, or 4.11%, respectively. Similarly, Chen and Liu discovered that glucose was the best carbon source to increase the capacity of *Pantoea* sp. S32 to solubilize phosphate was glucose. It was also shown that this strain was unable to grow and release phosphorus on a medium using sucrose and starch as carbon sources. In another work, Li et al. [21] investigated the use of glucose, sucrose, xylose, maltose, fructose, galactose, and mannitol as the carbon source and also showed that the greatest number of phosphates was liberated by *P. agglomerans* ZB during growth on glucose. Interestingly, when fructose was used as a carbon source, the pH level in the medium decreased insignificantly, from 7.0 to 6.2. We hypothesize that, in this case, the high efficiency of phosphate solubilization was achieved not only due to the release of organic acids, but also due to other mechanisms.

Another important macronutrient for bacterial growth is nitrogen [33]. When using ammonium sulfate ((NH_4_)_2_SO_4_), potassium nitrate (KNO_3_), yeast extract, peptone, and urea as the nitrogen source, P-solubilization efficiency of *P. brenneri* 3.5.2 achieved 74.56, 36.59, 40.7, 34.44, and 22.21%, respectively. The highest number of phosphates in the medium was observed when yeast extract was used as the source of nitrogen. However, a large number of phosphates were also found in the control flask (867.7 mg/L). This is likely due to the fact that yeast extract consists of a combination of proteins, peptides, amino acids, nucleic acids, vitamin B, carbohydrates, and other components. The exact chemical composition of the yeast extract varies and depends on the yeast cultivating conditions. The yeast extract contains phosphates [34], whose presence led to an increase in the background value of free phosphorus in the sample. Thus, we have shown that none of the studied nitrogen sources reduced the P-mobilizing activity of the *P. brenneri* 3.5.2, and the use of ammonium sulfate resulted in the highest efficiency of phosphate mobilization. Similarly, Walpola et al. [32] showed that *P. agglomerans* and *P. rodasii* release the highest amount of phosphorus from Ca_3_(PO_4_)_2_ while growing on ammonium sulfate. In addition, high values of free phosphates were obtained by replacing ammonium sulfate with ammonium chloride and ammonium nitrate, as well as calcium nitrate and potassium nitrate.

Environmental factors have a significant impact on the soil microbiome and biological processes carried out by microorganisms [35]. We investigated the effects of cultivation conditions (pH, temperature, rotation speed) on the efficiency of phosphate mobilization from Ca_3_(PO_4_)_2_ by *P. brenneri* 3.5.2. Our study suggested that *P. brenneri* 3.5.2 grew well at a broad range of temperatures, pH values, and rotation speeds. It achieved an optimal P-solubilizing rate in the following culture conditions: initial pH of the medium of 7.0, temperature of cultivation of 30 °C, and rotation speed of 200 rpm.

Phosphate-solubilizing bacteria have various mechanisms to increase the bioavailability of phosphorus in the soil [36]. One of the ways for bacteria to carry out P-solubilization is the secretion of mineral-dissolving complexes or compounds (siderophores, protons, hydroxyl ions, organic acids) [28]. Organic acids, which are low-molecular-weight compounds, are described as the main mechanism for inorganic phosphate solubilization [37]. In our study, an increase in the number of free phosphates in the medium correlated with a decrease of the medium pH: it dropped by more than half from 7.0 to 2.85. Saadouli et al. [38] demonstrated the ability of the *P. agglomerans* V8R67 strain to release phosphorus from tricalcium phosphate. The release of free phosphates also correlated with a decrease in the pH level of the medium from 7.8 to 4.65. The maximum decrease in the pH level of the medium was observed for *P. brenneri* strains 3.2 and 3.5.2, which were selected to study the production of organic acids. HPLC analysis detected different kinds and concentrations of organic acids in culture media supernatant. *P. brenneri* strains produced oxalic, malic, formic, malonic, lactic, maleic, acetic, and citric acids. In a recent study by Tahir et al. [39], the ability of two rhizobacteria (*Pantoea* sp. WP-5 and *Pseudomonas* sp. NN-4) to stimulate plant growth was investigated and production of various organic acids, such as acetic, citric, gluconic, succinic, and malic acids was shown. In the present study, *P. brenneri* strains were cultured on a medium containing glucose as a carbon source and produced formic and malic acids in high concentrations (>10 mM). We have shown that the spectrum and concentration of organic acids changed depending on the source of phosphorus in the medium. The maximum concentration of formic acid produced by *P. brenneri* 3.2 (11.2 mM) and *P. brenneri* 3.5.2 (10.1 mM) was observed during cultivation on a medium with Ca_3_(PO_4_)_2_. However, both strains did produce oxalic acid in these conditions. Heterogeneity in the synthesis of organic acids was also noted between strains.

During the HPLC-analysis of culture supernatants, in all experimental samples, we observed some peaks that were not present in the standards used, making it difficult to identify these compounds. Of particular interest was one peak with a release time of 6.6 (Figure S1), which corresponds to an unknown substance synthesized in relatively high concentration. We can exclude tartaric and malonic acids as possible compounds, because their retention times do not match this unknown peak. Moreover, based on the retention time, we can assume that the unknown acid is not aromatic. Additionally, the shape of the peak suggests that it either contains a keto group or an isolated double bond. According to other studies, many *Pantoea* strains are able to secrete gluconic or ketogluconic acids and have glucose dehydrogenase gene (*gcd*) and its cofactor pyrroloquinoline quinone (*pqq*) operon, responsible for that process [40]. Since we have identified *gcd* and *pqq* genes in the genome of *P. brenneri*, we expect that this unidentified peak corresponds to gluconic acid. In addition to the eight identified acids, these acids are potential candidates for further investigation.

The other way of increasing the bioavailability of soil phosphorus is related to the release of extracellular enzymes. The ability of microorganisms to solubilize phosphorus via this mechanism is mostly known for organic phosphates and is briefly described for inorganic phosphorus sources [2]. PSB secrete several groups of enzymes that are involved in phosphate metabolism. Namely, the phosphatases (phosphomonoesterases), which can be acidic or alkaline [41], belong to this enzyme group [42,43]. Both alkaline phosphatase (ALP) and acid phosphatase (ACP) activities were detected in *P. brenneri* strains growing on the medium containing hydroxyapatite, phosphorite, and tricalcium phosphate as the only source of phosphorus.

*P. brenneri* solubilization of the phosphate mineral employed different mechanisms: through the production of extracellular enzymes or by the release of organic acids, protons, and hydroxyl ions, which was confirmed by the presence of the corresponding genes in the genome of the bacteria. Among them are the gene for glucose dehydrogenase (*gcd*) and the gene encoding the cofactor of glucose dehydrogenase—pyrroloquinoline quinone PQQ (*pqqE*). *P. brenneri* also carries *pst* genes, accountable for phosphate binding, import, and transportation. Phosphonates represent an important source of bioavailable phosphorus. Many microorganisms possess catabolic pathways to degrade these molecules [44]. Enzymes involved in phosphonate catabolic pathways are conserved and encoded by ortholog genes in bacteria [45]. *P. brenneri* possesses *phn* genes, which are responsible for phosphonate solubilization, making these bacteria universal phosphate solubilizer. A full cluster of phosphate-specific transporters (PstSCAB) was identified in the genome of bacteria. *P. brenneri* has two phosphate-starvation-inducible (*psr*) genes, *psiE* and *psiF*, that react on the low level of P_i_ and activate inorganic phosphate transporter system such as phosphate-specific transporters (PstSCAB) as well as Pho-regulon [46].

In addition to wide-range phosphate solubilization ability, *P. brenneri* strains carry multiple PGP-traits such as IAA and siderophores production, N-fixation ability, cellulase and protease activity, and antagonistic features [17]. Due to these properties, *P. brenneri* strain 3.5.2 was tested for wheat plant growth promotion in the laboratory experiments. Our results showed that wheat seeds inoculated with *P. brenneri* 3.5.2 had increased root and shoot length compared to control. This may have occurred due to the production of IAA.

Potato is grown in more than 150 countries all around the world, and total production has reached up to 359.1 million tons [47]. We studied the inoculation of potato tuber with *P. brenneri* strains in greenhouse experiments. Inoculation with *P. brenneri* strains 3.1 and 3.5.2 showed maximum increases in stem height, and the tuber yield of potato plants when treated with *P. brenneri* 3.2 was 33.2 g/plant versus 28 g/plant for uninoculated control plants.

## 5. Conclusions

The use of modern biotechnology methods opens up new opportunities for realizing the genetic potential of rhizosphere microorganisms. *P. brenneri* strains have a unique feature to possess high dissolving capacity for both inorganic and organic hardly soluble soil phosphorus and promote plant growth due to their multiple PGP-treats. The obtained results suggest that *P. brenneri* strains with a wide range of phosphate solubilizing activity and multiple PGP-features have potential as biofertilizers for improving plant growth and yield. Further studies are needed to evaluate their activity directly in the soil systems, study their performance under field conditions, and assess their impact on soil microbial communities.

## Figures and Tables

**Table 1:** Concentration of produced organic acids by *P. brenneri* 3.2 and *P. brenneri* 3.5.2 strains on liquid NBRIP containing different P-sources.

Strain	P-Source	Organic Acids (mM)							
		Oxalic	Malic	Formic	Malonic	Lactic	Maleic	Acetic	Citric
*P. brenneri* 3.2	Hydroxyapatite	-	1.02 ± 0.3	6.22 ± 1.5	1.73 ± 0.7	-	0.03 ± 0.2	-	-
	Phosphorite	-	7.56 ± 0.4	7.24 ± 3.3	-	0.59 ± 0.27	0.02 ± 0.001	0.59 ± 0.17	0.16 ± 0.04
	Tricalcium phosphate	-	12.78 ± 1.01	11.3 ± 3.66	-	1.37 ± 0.4	0.02 ± 0.001	0.95 ± 0.3	0.15 ± 0.04
*P. brenneri* 3.5.2	Hydroxyapatite	1.95 ± 0.25	13.76 ± 1.17	8.7 ± 3.2	0.47 ± 0.05	2.06 ± 0.96	-	0.75 ± 0.27	0.13 ± 0.04
	Phosphorite	0.46 ± 0.07	9.6 ± 0.66	7.54 ± 2.00	-	1.62 ± 0.3	0.03 ± 0.003	1.00 ± 0.03	0.17 ± 0.03
	Tricalcium phosphate	-	11.54 ± 0.82	10.11 ± 2.9	0.28 ± 0.02	1.35 ± 0.1	0.03 ± 0.001	0.5 ± 0.07	0.16 ± 0.03

**Table 2:** Genes related to phosphorous uptake and solubilization.

Locus Tag	Gene	Product	Pathway
KKD31040 KKD31973	*gcd*	quinoprotein glucose dehydrogenase	Gluconate production
KKD33374	*pqqB*	pyrroloquinoline quinone biosynthesis protein PqqB	
KKD33373	*pqqC*	pyrroloquinoline quinone biosynthesis protein PqqC	
KKD33372	*pqqD*	pyrroloquinoline quinone biosynthesis peptide chaperone PqqD	
KKD33371	*pqqE*	pyrroloquinoline quinone biosynthesis protein PqqE	
KKD33370	*pqqF*	pyrroloquinoline quinone biosynthesis protein PqqF	
KKD32511	*ppx*	Exopolyphosphatase	Degradation of inorganic polyphosphates
KKD32512	*ppk*	Polyphosphate kinase	
KKD31812	*ppa*	Inorganic pyrophosphatase	
KKD34216	*psiF*	Phosphate starvation-inducible protein PsiF	
KKD31910	*psiE*	Phosphate starvation-inducible protein PsiE	
KKD34188 KKD30886	*phyK*	3-phytase	Organic phosphates mineralization
KKD33729	*agpP*	glucose-1-phosphatase AgpP	
KKD32984	*phoA*	alkaline phosphatase	
KKD30737	*pstS*	phosphate transport system substrate-binding protein	Phosphate transport
KKD30736	*pstC*	phosphate transport system permease subunit PstC	
KKD30735	*pstA*	phosphate transport system permease subunit PstA	
KKD30734	*pstB*	phosphate transport system ATP-binding protein	
KKD32884	*phnF*	phosphonate metabolism transcriptional regulator	Degradation of phosphonates
KKD32885	*phnG*	phosphonate C-P lyase system protein	
KKD32887	*phnI*	carbon-phosphorus lyase complex subunit PhnI	
KKD32888	*phnJ*	carbon-phosphorus lyase complex subunit PhnJ	
KKD32889	*phnK*	phosphonate C-P lyase system protein	
KKD32890	*phnL*	phosphonate C-P lyase system protein	
KKD32891	*phnM*	phosphonate metabolism protein PhnM	
KKD32892	*phnN*	ribose 1,5-bisphosphate phosphokinase PhnN	
KKD32894	*phnC*	phosphonate transport system ATP-binding protein	
KKD32895	*phnD*	phosphonate transport system substrate-binding protein	
KKD3289	*phnE*	phosphonate ABC transporter permease	

## Data Availability

The data presented in this study is contained within this article.

## Supplementary Materials

The following supporting information can be downloaded at: https://www.mdpi.com/article/10.3390/microorganisms11051136/s1, Figure S1: Organic acid production by *P. brenneri* 3.2 and *P. brenneri* 3.5.2 on liquid NBRIP media with different P sources.
