# Supplementary material for: Phosphate Solubilization and Plant Growth Promotion by Pantoea brenneri Soil Isolates
Source: Microorganisms. 2023 Apr 27;11(5):1136. doi: 10.3390/microorganisms11051136 (PMC10221785; doi:10.3390/microorganisms11051136)
Supplement: Supplementary file 1 [file microorganisms-2331964-supplementary.pdf › microorganisms-2331964-supplementary.pdf]

Supplementary Material

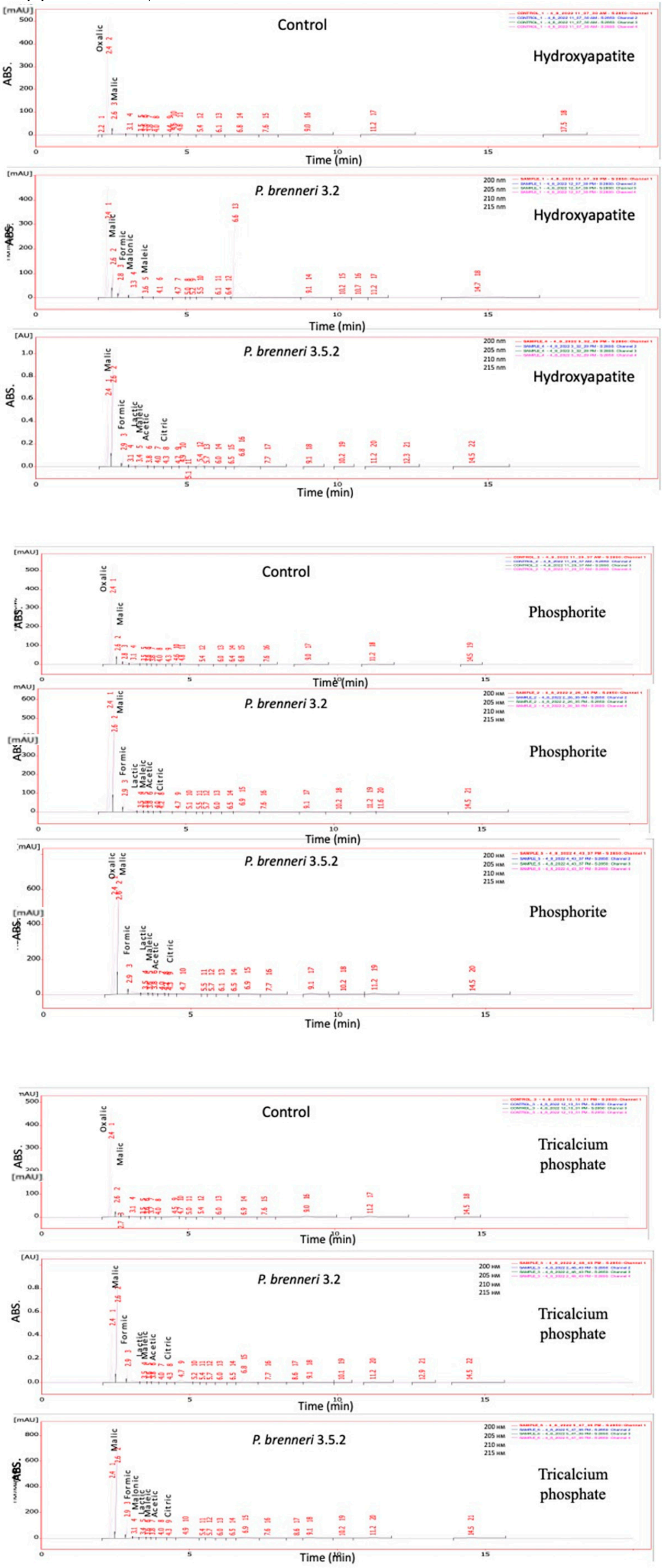

Figure S1. Organic acid production by *P. brenneri* 3.2 and *P. brenneri* 3.5.2 on liquid medium NBRIP with different P sources: A – hydroxyapatite, B – phosphorite, C – tricalcium phosphate.
